# Knowledge, Attitudes, and Practices of Speech Language Pathologists in India about Telerehabilitation Services during the COVID-19 pandemic

**DOI:** 10.1590/2317-1782/20212021193

**Published:** 2022-05-11

**Authors:** Gagan Bajaj, Sudhin Karuppali

**Affiliations:** 1 Department of Audiology and Speech Language Pathology, Kasturba Medical College, Mangalore, Manipal Academy of Higher Education – MAHE – Manipal, Karnataka,, India.

**Keywords:** Attitude, COVID-19, Knowledge, Practice, Speech Therapy, Telerehabilitation

## Abstract

**Purpose:**

The global impact of the COVID-19 pandemic has opened opportunities for service providers and patients to continue with clinical services in certain extraordinary settings and circumstances. Telerehabilitation in the field of speech language pathology in India is still at its infancy, with a majority of the Speech Language Pathologists (SLP) accustomed with the conventional face-to-face system of service delivery. The present study aims to gather the knowledge, attitudes, and practices (KAP) of SLPs in India regarding telerehabilitation services during the pandemic.

**Methods:**

The study was conducted in three phases: phase I involved the development and validation of a questionnaire to explore the KAP of SLPs regarding telerehabilitation services. The items were framed based on a Likert rating scale *(strongly agree, agree, neutral, disagree, and strongly disagree),* yes-no-maybe format, open-ended, and multiple-choice format. Phase II involved data collection, while phase III involved data analysis. Descriptive statistics was done to derive the frequency and percentage for discrete variables and mean and SD for continuous variables.

**Results:**

Many SLPs feel underprepared in their technical knowledge and skills needed for telerehabilitation. Furthermore, a majority of the SLPs also did report patients to be relatively lesser motivated and satisfied with tele practices due to issues that are discussed in the paper.

**Conclusion:**

This study is an initial attempt to touch upon the fabric of telerehabilitation services delivered by SLPs of India. Future studies are directed to study the technical, professional, and personal issues encountered during telerehabilitation services specifically pertaining to specific communication disabilities.

## INTRODUCTION

The Coronavirus disease (COVID-19) pandemic started due to a highly infectious disease caused by a newly discovered coronavirus in December 2019 in Wuhan, China. The disease gets transmitted through droplets that are generated when an infected person exhales, sneezes, or coughs. The global impact of COVID-19 pandemic has called upon for desperate and immediate strategic, innovative, and adaptive methods for health-care service delivery. The use of interactive telemedicine has been evident as early as 1959 in the transmission of neurological findings^(1)^, with the field of telemedicine in terms of both healthcare delivery and technology evolving with time^(2)^. With the usage of wireless broadband technology progressing, and the internet becoming a universal phenomenon, patient education with real-time video and audio consultations have become a reality. Such state-of-the-art consultation methods use existing computing devices belonging to service providers and receivers, making it convenient for the former to gather clinical data for effective service delivery^(2)^, thereby overcoming barriers of geographical distance.

Speech language pathologists (SLPs) are among the highly vulnerable health professionals, who are associated with individuals with communication and swallowing disabilities, mostly requiring to be in close physical proximity during diagnostic and interventional based procedures. Patients with communication disabilities in India have limited access to speech and language services due to the shortage of service providers^(3)^. This has resulted in the urgent need to meet the demands of these individuals who seek such clinical services in their respective regions across India. With access to widespread internet connectivity throughout the country, there is emerging scope for SLPs to engage in telepractice to offer their clinical services to the public^(4)^. The American Speech-Language-Hearing Association (ASHA) adopted the term telepractice as an alternative to telehealth or telemedicine to evade confusion that such services are provided only by medical professionals. ASHA states telepractice as the application of telecommunications technology to the delivery of professional services delivered by audiologists and SLPs at a distance by connecting client to clinician or clinician to clinician for consultation, assessment, and/or intervention. However, the American Telemedicine Association does include all services delivered by SLPs and audiologists to be included under a broader generic term called telerehabilitation. Research reports 40% of the tele-based services conducted on communication disabilities, primarily focused on assessment or intervention, with the largest number of published studies on the same being reported from the United States of America (32%), followed by Australia (29%)^(5)^. The applications of telerehabilitation in the field of speech language pathology have been reported in the areas of stuttering, swallowing dysfunction, speech and language disorders in children, adult neurogenic language and speech disorders, laryngectomy, and voice disorders^(6)^. Mohan et al.^(4)^ carried out a survey among 205 Indian SLPs and audiologists about telepractice services. They reported that majority of the SLPs and audiologists in India were aware of telepractice as a mode of service delivery but only one-tenth of the surveyed participants actively engaged in telepractice services. As reported in their study, the disorders which were being dealt in telepractice mode by the professionals involved childhood aphasia, specific language impairment, motor speech disorders in children, dysphagia in children and adults, auditory processing disorders and vestibular disorders. A Korean study done on the perception of SLPs on telepractice found roughly only 6% of the respondents to practice telerehabilitation services^(7)^. The authors of this study also reported the poor acceptance of telerehabilitation services by SLPs who have more clinical experience, thereby considering such online delivery services as incomparable to the traditional therapy. A Croatian study^(8)^, surveyed 255 SLPs on the perceptions and applications of telepractice in SLP settings during the COVID-19 pandemic and found 71% of SLPs to have offered telerehabilitation services, out of which only 54% admitted being satisfied with the delivery mode. Contrastively, a very high level of satisfaction (ranging from 93.7-99%) was reported by patients who received telerehabilitation services from speech language pathologists, physical therapists, and occupational therapists^(9)^.

Telerehabilitation has opened opportunities for service providers and patients to continue with the clinical services in certain extraordinary settings and circumstances. Advantages of telerehabilitation services might be promising for continuing undisrupted clinical services during a situation like a natural disaster^(10)^. Prior to the onset of the present pandemic, only about 10% of SLPs reported indulging in telerehabilitation services or training as compared to greater than 60% SLPs either getting trained or practicing telerehabilitation around May 2020(11). However, many SLPs expressed that sudden transition to telerehabilitation services since COVID 19 pandemic has left them feel underprepared and overwhelmed^(11)^. The sudden uptake of telerehabilitation services by SLPs in India due to the pandemic might be challenging. India is a populous country catering to a large number of speech and hearing disabilities, having more than 3500 registered audiologists and SLPs. Telerehabilitation in the field of speech language pathology in India is still at its infancy, with a majority of the SLP professionals accustomed with the conventional face-to-face system of delivering speech and language therapy. Researchers^(3)^ do realize that Indian SLPs are naïve about the logistics of using online resources maintaining patient privacy, providing accessibility, and protecting data, indicating the need to address such concerns before the commencement of telerehabilitation-based services in a larger scale. Moreover, it remains unclear if the different stakeholders possess appropriate skills and technological support to adjust to this sudden shift. Therefore, the present study aims to gather the knowledge, attitudes, and practices of the SLPs in India regarding telerehabilitation services during this COVID-19 pandemic.

## Methods

The current study was a self-reported internet based study following a cross-sectional study design and a convenience sampling method. It was approved by the Institutional Ethical Committee (IECKMCMLR-09/2020/261) of Kasturba Medical College, Mangalore, Manipal Academy of Higher Education.

### Participants

The developed survey was sent out to a total of 102 participants out of which all responded. The mean (SD) age of the participants was 28.5 (5.0), who were within the age range of 21 and 68 years. Table 1 depicts the demographic details of the participants.

**Table 1:** Demographic details of participants

Demographic Variables	Characteristics	Frequency (n)	Percentage (%)
Gender	Males	10	9.80
	Females	91	89.21
	Prefer not to say	1	0.98
Qualification	BASLP	23	22.55
	MASLP	56	54.90
	M.Sc. (SLP)	16	15.69
	Ph.D.	4	3.92
	Others	3	2.94
Clinical experience (in years)	<1	4	3.92
	1-5	58	56.86
	6-10	22	21.57
	11-15	9	8.82
	16-20	4	3.92
	>20	5	4.90
Work setting	Private	53	51.96
	Hospital	33	32.35
	Institution	23	22.55
	Rehabilitation center	8	7.84
	Others	10	9.80

BASLP- Bachelor of Audiology and Speech Language Pathology;

MASLP- Master of Audiology and Speech Language Pathology;

M.Sc. (SLP)- Master of Science in Speech Language Pathology;

Ph.D.- Doctor of Philosophy;

n- number of participants belonging to the specified category

### Procedure

The study was conducted in three phases wherein phase I involved the development and validation of the questionnaire; phase II involved data collection; and phase III involved data analysis.

### Phase I: Development and validation of the questionnaire

The questionnaire for the study was designed to explore the Knowledge, Attitudes, and Practices of SLPs regarding telerehabilitation services during the COVID-19 pandemic. The developed questionnaire was content validated by three SLPs with a minimum of 5 years of clinical and research experience. Each of the prepared items of the questionnaire were rated by the experts using a Likert rating scale of *very appropriate (score 4), appropriate (score 3), can’t say (score 2), inappropriate (score 1),* and *very inappropriate (score 0).* An average of the scores for each item given by the three experts was calculated, with 4 being the maximum average score and 0 being the minimum average score possible. An average score of 3.8 (Content validity index of 0.95) was obtained, indicating an excellent content validity of the developed questionnaire. Suggestions and comments were incorporated from each of the experts. The final questionnaire which was designed in English did comprise of 24 items pertaining to the Knowledge, Attitudes and Practice of SLPs on the conduction of telerehabilitation services, and 7 items pertaining to the demographic details of the respondents. Responses for these items were collected through Likert rating scale *(strongly agree, agree, neutral, disagree, and strongly disagree),* yes-no-maybe format, open-ended, and multiple-choice format.

### Phase II: Data collection

The final questionnaire was entered into an online survey creator (Microsoft Form – part of Office 365) with a common link made available for the same. The link was then shared via WhatsApp (which is a cross-platform messaging service) to multiple speech and hearing professional groups. All participants were explained about the purpose of the study. The survey began with a brief description of the study, followed by an informed consent which was taken before the participants participated in the study. Participation towards this study was purely on voluntary basis. The data collection was carried out between 4^th^ and 20^th^ of July 2020. The average time taken to complete the questionnaire was 9 minutes. All responses were automatically saved online and could only be accessed by the authors. The questionnaire was administered during Unlock 2 process (which lasted between 1^st^ and 31^st^ July) as per the guidelines imposed by the Ministry of Home Affairs and the National Disaster Management Authority of India.

### Phase III: Data analysis

Data analysis was performed using SPSS 16. Descriptive statistics was done to derive the frequency and percentage for discrete variables and mean and SD for continuous variables.

## Results

### Knowledge of Telerehabilitation Services

The questionnaire comprised of three questions pertaining to the knowledge of SLPs about telerehabilitation services. Participants were asked about their familiarity with telerehabilitation services before the pandemic, changes in their technical knowledge essential for telerehabilitation with pandemic, and their participation in any sort of technical orientation for this purpose. These items were rated by the participants as either ‘yes’, ‘no’ or ‘maybe’. The findings of these results have been summarized in the Table 2.

**Table 2:** Knowledge related measures related to telerehabilitation services

	Responses	Participants
		n (%)
Were you familiar/or practiced telerehabilitation services before the occurrence of COVID-19 pandemic?	Yes	46 (45.09)
	No	56 (54.90)
	Maybe	NA
Have your technical know-how pertaining to setting up and conducting telerehabilitation services improved after the occurrence of the COVID-19 pandemic?	Yes	74 (72.55)
	No	5 (4.90)
	Maybe	23 (22.55)
Did you have any orientation/training on online consultation?	Yes	36 (35.29)
	No	66 (64.71)
	Maybe	NA

n- number of participants belonging to the specified category;

NA- Not applicable

### Attitudes towards Telerehabilitation Services

This section of the questionnaire comprised of eleven questions pertaining to the attitudes of the participants towards telerehabilitation services. Seven of these questions were rated by the participants to share their own feelings and perspectives about telerehabilitation, whereas in the rest of the four items, participants expressed their perception about what their clients/patients seem to feel about the telerehabilitation services. Three of these four questions were rated by the participants, and one of the question pertaining to expectations of their patients from telerehabilitation services was collected in an open ended format. The attitude responses obtained for the rating based questions have been described in terms of frequency and percentages in Table 3.

**Table 3:** Attitude related measures (agreeability) related to telerehabilitation services

**Attitudes pertaining to self**	**Strongly agree n (%)**	**Agree n (%)**	**Neutral n (%)**	**Disagree n (%)**	**Strongly disagree n (%)**	**Yes n(%)**	**No n(%)**	**Not sure n(%)**
I feel it convenient to use online platforms for providing telerehabilitation services during the COVID-19 pandemic	23 (22.55)	44 (43.14)	29 (28.43)	3 (2.94)	2 (1.96)			
I feel confident to use online platforms for providing telerehabilitation services during the COVID-19 pandemic	19 (18.63)	46 (45.10)	30 (29.41)	6 (5.88)	0 (0)			
I feel that my clinical skills are becoming questionable after beginning telerehabilitation services.	5 (4.90)	10 (9.80)	23 (22.55)	43 (42.16)	20 (19.61)			
I feel that the telerehabilitation services are not as effective as direct consultation.	23 (22.55)	38 (37.25)	21 (20.59)	14 (13.73)	5 (4.90)			
I feel incomplete after providing telerehabilitation services.	9 (8.82)	31 (30.39)	22 (21.57)	32 (31.37)	7 (6.86)			
I feel concerned about the privacy issues involved while using an online platform for telerehabilitation services.						49 (48.04)	29 (28.43)	23 (22.55)
I plan to continue providing telerehabilitation services even after the COVID-19 pandemic ends.						45 (44.12)	25 (24.51)	31 (30.39)
**Attitudes pertaining to patient’s perceptions**								
I feel that my patients have lost their persistence and motivation to attend speech therapy after the occurrence of COVID-19 pandemic	5 (4.90)	25 (24.51)	35 (34.31)	31 (30.39)	5 (4.90)			
I feel that my patients feel that it is not worth spending money on services that are provided in an online platform compared to face-to-face?	7 (6.86)	21 (20.59)	29 (28.43)	35 (34.31)	9 (8.82)			
My patients feel concerned about the privacy issues involved while using any online platform for telerehabilitation services.						26 (25.49)	46 (45.09)	29 (28.43)

When asked to describe the nature of expectations their patients had from telerehabilitation services, most of the participants shared that their patients expected the telerehabilitation services to possess similar quality as face to face therapy (20.8%) ensuring equivalent progress in patient in status like face to face therapy (13.1%). Some of the other patient expectations from telerehabilitation services as expressed by the participants were: to receive appropriate professional guidance and coaching (8.7%); to include novel and interactive activities during therapy to keep the pediatric patients engaged (5.4%); to optimize the frequency and duration of the therapy sessions (3.2%); and to ensure appropriate participations of the parent/caregiver during the telerehabilitation sessions (2.1%). Few participants also expressed that their patients expected a better feedback and counseling mechanism during the online sessions with some expecting the therapy charges to be relatively lesser for online mode of delivery.

### Practices towards Telerehabilitation Services

In this section of the questionnaire, participants were first asked to choose the online platforms from the given list that were being actively used by them to deliver the telerehabilitation services since the onset of pandemic. Table 4 depicts the percentage of participants using each of the online platforms.

**Table 4:** The percentage of participants using different platforms to provide telerehabilitation services

Online platforms	Percentage of participants
1. WhatsApp	59%
2. Zoom	70%
3. Google handouts/Duo	19%
4. Skype	29%
5. Face time	5%
6. GoToMeeting	6%
7. Facebook Messenger	4%
8. Others	22%

**Figure 1:**
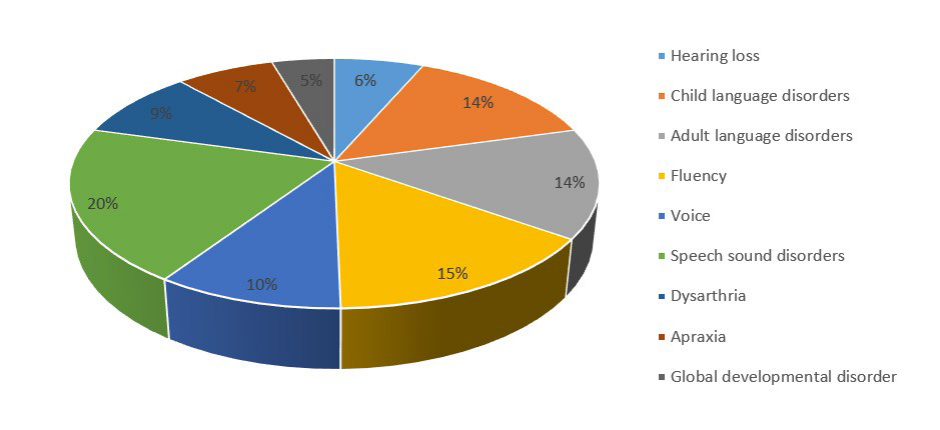
The percentage of the type of communication disorders SLPs provide telerehabilitation services

The next few questions in this section provided an insight into the nature of clientele managed by the participants through telerehabilitation mode during the pandemic. Here, 41.18% SLPs expressed that they found it difficult to convince the patients/caregivers to stay motivated during the course of telerehabilitation services while rest of the SLPs were either unsure or did not report this problem. Most of the SLPs also reported that it was easier to convince the existing patients for telerehabilitation services (62.75%) as compared to the new patients. When asked about the age group of their telerehabilitation clientele, most SLPs reported that major share of their client load through telerehabilitation mode were children (64%) followed by geriatrics (22%), adolescents (7%) and were adults (6%). Participants were further asked to list the communication disorders that they had been managing through telerehabilitation mode during the pandemic. Figure 1 describes the proportion of communication disorders as reported by the participants.

Participants were then asked to identify the communications disorders which they found to be challenging to handle through the online mode. A total of 16% of SLPs did not respond to this question. Out of those who responded, 43% of them found ASD to be the most challenging communication disorder to manage through online mode, followed by ADHD (14%), feeding/swallowing related disorders (13%), speech sound disorders (12%), hearing impairment (10%). Management of other communication disorders like fluency disorders, voice disorders, dysarthria were found to be least challenging (4% - 7%). In the later section of the ‘practices’ domain, SLPs were asked to share certain logistic aspects of telerehabilitation services. Regarding the professional charges of the telerehabilitation services, 38% of the SLPs expressed that they continued to maintain the same charges for telerehabilitation services; 30% of SLPs shared that they charged lesser professional charges for telerehabilitation services as compared to face to face therapy, and 7% SLPs reported increased professional charges through online mode. Around 25% of SLPs chose not to comment on this.

**Figure 2:**
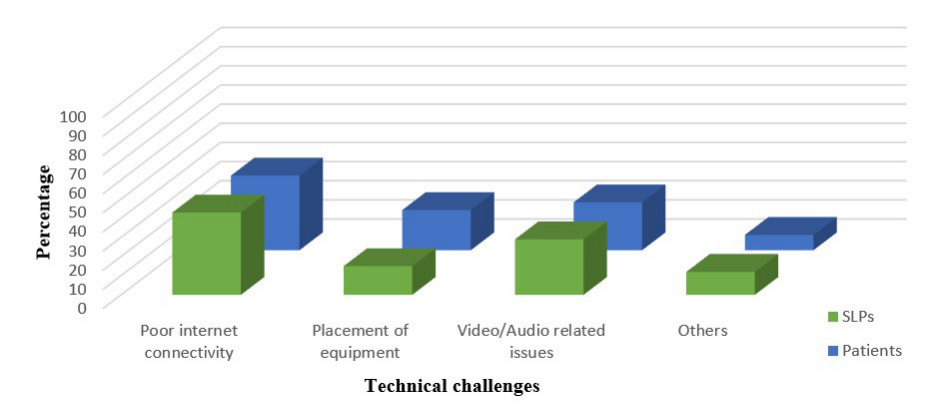
The percentage of SLPs and their patients having different technical challenges during telerehabilitation services

Participants were then asked to list the practical challenges faced by them to plan and execute telerehabilitation services with their patients. Majority of the participants expressed that interference/distraction during screen time (61%) was the most challenging aspect in telerehabilitation services. Many SLPs reported that guiding the patient/caregiver during the online session (48%), visual fatigue for the clinician and patients due to prolonged screen time (48%), and scheduling a convenient time suitable to the therapist and the patient (44%) were some of the significant challenges. Positioning/seating arrangement of the patient during the telerehabilitation session was also reported to be an important practical challenge by some of the SLPs (39%). Besides the practical challenges, participants also reported certain technical challenges faced by them and their patients during the telerehabilitation services. These technical challenges have been illustrated in Figure 2.

## Discussion

The current study was planned to understand the knowledge, attitudes, and practices of SLPs in India pertaining to the telerehabilitation services provided during the COVID-19 pandemic. Speech language pathologists between a wide age range (21-68 years) did participate in the survey, with a major bulk of the population being between 20 and 40 years of age. This may not be a surprising trend, as the majority of technology-savvy consumers have been the younger population when compared to older individuals^(12)^. A similar pattern was evident in the clinical experience of the participants in the survey. Assuming that one may complete his/her post-graduation at around 22 or 23 years of age, and immediately beginning his/her professional career, one would gather a clinical experience of around 15 years by the time he/she reaches his late 30s. This was clearly reflected in the obtained responses, with almost 91% of the respondents being below 40 years of age. The participants did represent professionals from all types of work settings (private clinics, hospitals, institutes, and rehabilitation centers). When considering the gender distribution of the use of telerehabilitation services, a total of 89% of the participants were females when compared to males (10%), indicating a profound inclination towards the female gender, which is well established in the field of speech and language sciences^(13)^.

### Knowledge of Telerehabilitation Services

Around 45% of the participants did report of being familiar or practice telerehabilitation services during the pre-pandemic period, with the majority (52%) of the participants being private practitioners. However, 55% of the participants who were unfamiliar or lacked digital knowledge and competency^(8,14)^, or did not practice telerehabilitation services, were mostly professionals practicing at institutes, hospitals, and rehabilitation centers, wherein face-to-face speech and language therapy is generally enforced upon to maintain traditional standards. Though a large number of participants (73%) improved upon their technical know-hows of setting up and conduction of telerehabilitation services after the occurrence of the COVID-19 pandemic, a small majority (23%) were still unsure of the same. This could probably be since 66% of the participants were not oriented nor trained for the conduction of telerehabilitation, which is a major contributing factor, which has been previously reported as well^(15)^. These findings direct for a comprehensive training for SLPs for the conduction of telerehabilitation services which was also previously recommended by Mohan et al^(4)^. Kim et al^(7)^ also found Korean SLPs to exhibit an increased willingness to undergo such training programs regardless of their age, education, or career.

### Attitudes towards Telerehabilitation Services

Although the use of tele-based services was popularly becoming the new norm for service delivery among SLPs in India during the COVID-19 pandemic, it did not come without the cost of privacy. A total of 48% of the participants were concerned with their privacy using such delivery methods, although 28% of them were unconcerned. However, this was not the same when considering the patients, where only 25% did feel threatened by privacy issues than a vast majority (45%) who remained unthreatened by the same. Probably, since a majority (55%) of the SLPs who were unfamiliar/or did not practice telerehabilitation services, were compelled to initiate the same, would have resulted in an increase in the concerns they had towards privacy related issues. While, the patients who were the primary stakeholders of the healthcare delivery, would have been more worried of missing out on their regular therapy sessions due to the imposed lockdown, cause of which privacy would not have been their primary concern. However, 25% of the participants felt their patients were concerned. In a scoping review^(16)^, the authors did report one of the challenges faced while balancing privacy and confidentiality, and the patient needs, was the commercialization of patient data. It was therefore recommended to use participatory health-enabling technologies to ensure the protection of patient privacy and confidentiality^(4,16)^, and having strict guidelines and polices for secure service delivery^(4)^. When asked about the convenience and confidence felt while using online platforms for telerehabilitation during the pandemic, 43-45% of the participants did agree to the same, and also did plan on continuing tele-based services even after the COVID-19 pandemic ends. Such a decisional change in service delivery, realizing the use of tele mode to be encouraging and promising have been reported in other research as well^(17)^, with telerehabilitation being found to be as effective as face-to-face therapy^(18)^.

Telerehabilitation services are found to be a valid and reliable vehicle^(19)^ for delivering communication and swallowing rehabilitation related services^(4)^, with a capacity to enhance functional outcomes by enabling generalization of intervention effects within the person's daily environment, permitting monitoring of the same on a long-term basis^(6)^. It was also reported that clinicians can optimize the intensity, duration, and timing of therapy when using telerehabilitation services, which was not possible when following the traditional face-to-face treatment protocols^(20)^. However, in the current survey around 28% of the participants reported to be neutral to this feeling. Although 23-37% of the participants agreed that telerehabilitation services lacked acceptance^(14)^ and were not as effective as direct consultation^(7,8)^, a small majority (5-14%) were not of the same opinion.

When asked questions pertaining to their patient’s persistence and motivation levels to attend telerehabilitation services after the occurrence of the COVID-19 pandemic, around 5% of the participants strongly agreed that patient’s did lose their persistence and motivation when compared to the pre-pandemic period, with 7% of them feeling less worthy of spending money on such services when compared to the traditional face-to-face/direct therapy, which was noted in a previous study as well^(21)^. However, 29-34% of the participants were neutral to this perception. Considering their clinical expertise after beginning telerehabilitation services, 62% of the participant’s felt no change in the same, though 15% reported otherwise. Around 39% of the participants were left with the feeling of being incomplete after providing telerehabilitation services, mostly because of this sudden change in mode, and also probably wanting a direct interaction. Such use of hybrid methods to maintain service standards has been previously reported^(22)^. But 37% of the participants were not of the same opinion.

### Practice towards Telerehabilitation Services

When considering the type of online platforms used by the participants for delivering telerehabilitation, a major proportion of the participants used Zoom (70%) and WhatsApp Messenger (59%). Other studies also did report the use of video-based platforms such as Skype^(23)^ and Face time^(24)^ which was used in the current study as well. On examining the responses obtained by the participants pertaining to the type of population who were provided with telerehabilitation services, results did indicate the pediatric population to be the biggest (64%) stakeholders^(25)^. This is however not uncommon due to the fact that communication disabilities in children may arise from a result of a variety of conditions^(26)^. The pre-school group make up a majority of the pediatric clinical population who require speech and language therapy services, as currently school teachers are getting equipped in the identification of communication and learning challenges in young students. In the current study, irrespective of the age of the patient, it was noted that 20% of telerehabilitation services were provided for patients with speech sound disorders, followed by 14-15% for child language disorders, adult language disorders, and fluency related disorders. Patients with dysarthria and apraxia, and global developmental disorders made up 5-9% of the population receiving telerehabilitation services. Since the participants in the current study were primarily private practitioners, and professionals working in hospitals and institutes, the type of disorders catered by them are diverse, compared to rehabilitation centers which generally focuses on handling specific disabilities. Patients with autism spectrum disorders (ASD) were the most common (30%) and most challenging (43%) disability while providing telerehabilitation services, possibly due to the wide range of coexisting disorders and problems observed in this population^(27)^. The current study also reports of providing telerehabilitation services for individuals with ADHD, voice disorders, intellectual disabilities, feeding/swallowing disorders, and hearing loss. Although the SLPs perception of the benefit of telerehabilitation across disorders was not the objective of the current study, results did indicate the latter^(9)^ to be more challenging.

Considering India being a relatively less developed country, numerous challenges and barriers were faced during the telerehabilitation. A few of the challenges reported by Rao and Yashaswini^(3)^ were service delivery policy, evidence-based practice measures, privacy and confidentiality on using e-platforms, ethical considerations, cost-benefit, and risk analysis, and data protection. Regina Molini-Avejonas et al^(5)^ claimed that the barriers pertaining to tele practice services generally does pertain to technology, acceptance, training, recognition, and therefore the regulation of such services requires to be addressed immediately. Similar to the other studies reported in literature, the technical challenges encountered during telerehabilitation services in the current study (by both SLPs and their patients) included slow internet connectivity (39-43%)^(28)^, video/audio issues (25-29%)^(28)^, and equipment placement (15-21%)^(29)^. Such technical issues encountered during telerehabilitation services by the participants of the current study would have influenced a majority of the SLPs (38%) to retain the same charges, and some (30%) even to decrease the same. Such issues probably would have resulted in the SLPs (41%) failing to convince the patients/caregivers to stay motivated during the course of the telerehabilitation service, as lack of patient participation and poor adherence to telerehabilitation services have been previously reported as well^(30)^, which can be attributed to the tele sessions being inconvenient and time consuming, especially between their regular busy work schedule^(8)^. A secure professional and personal bond created between the SLP and the patient, would have helped the SLPs (63%) to continue their services (via tele) in spite of the pandemic situation. Other major practical challenges faced by SLPs while providing telerehabilitation services may be a result of this service delivery approach which is new to almost 50% of the participants in the current study. Keck and Doarn^(22)^ does suggest SLPs to use hybrid (conventional and tele-based) methods to maintain service standards, as the use of advanced technology has limitations in the application of telehealth. They also indicated that although technological hardships were not reported as the primary cause of discontinuation of telehealth services by the practitioners, visual and audio disturbances were largely related with videoconferencing.

## CONCLUSION

The COVID-19 pandemic has resulted in a surge of telerehabilitation services delivered by SLPs across the world. It therefore becomes imperative to start exploring the efficacy of this service delivery method which is now flourishing. Moreover, in a country like India, where in-person SLP services are yet to become accessible across various geographical locations, the scope of telerehabilitation SLP services is relevant even beyond the present pandemic. The current study is an initial attempt to touch upon the fabric of telerehabilitation services delivered by SLPs of India. Future studies are directed to study the technical, professional, and personal issues encountered during telerehabilitation services specifically pertaining to specific communication disabilities, thereby helping shape standard guidelines that may be beneficial for the implementation of telerehabilitation services by SLPs.
